# Listening to your partner: serotonin increases male responsiveness to female vocal signals in mice

**DOI:** 10.3389/fnhum.2023.1304653

**Published:** 2024-01-24

**Authors:** Kayleigh E. Hood, Laura M. Hurley

**Affiliations:** ^1^Hurley Lab, Department of Biology, Indiana University, Bloomington, IN, United States; ^2^Center for the Integrative Study of Animal Behavior, Indiana University, Bloomington, IN, United States

**Keywords:** inferior colliculus, serotonin, vocalization, courtship, USV, squeak

## Abstract

The context surrounding vocal communication can have a strong influence on how vocal signals are perceived. The serotonergic system is well-positioned for modulating the perception of communication signals according to context, because serotonergic neurons are responsive to social context, influence social behavior, and innervate auditory regions. Animals like lab mice can be excellent models for exploring how serotonin affects the primary neural systems involved in vocal perception, including within central auditory regions like the inferior colliculus (IC). Within the IC, serotonergic activity reflects not only the presence of a conspecific, but also the valence of a given social interaction. To assess whether serotonin can influence the perception of vocal signals in male mice, we manipulated serotonin systemically with an injection of its precursor 5-HTP, and locally in the IC with an infusion of fenfluramine, a serotonin reuptake blocker. Mice then participated in a behavioral assay in which males suppress their ultrasonic vocalizations (USVs) in response to the playback of female broadband vocalizations (BBVs), used in defensive aggression by females when interacting with males. Both 5-HTP and fenfluramine increased the suppression of USVs during BBV playback relative to controls. 5-HTP additionally decreased the baseline production of a specific type of USV and male investigation, but neither drug treatment strongly affected male digging or grooming. These findings show that serotonin modifies behavioral responses to vocal signals in mice, in part by acting in auditory brain regions, and suggest that mouse vocal behavior can serve as a useful model for exploring the mechanisms of context in human communication.

## Introduction

During vocal communication, contextual cues play an important role in conveying meaning for both human and animal listeners. Cues that add information may be acoustic, such as the structure of vocal signals that convey the emotional state of the speaker or urgency of a message (e.g., Zinchenko et al., 2018; Christensen et al., 2019; Coss et al., 2019; Schneider et al., 2021). Important contextual cues can also occur in different sensory modalities. These include visual cues like facial expression or gestures that can increase the understanding of a speaker’s intent under challenging listening conditions such as in background noise (Moradi et al., 2013), or convey nuances like irony or sarcasm (Sumby and Pollack, 1954; Sommers et al., 2005; Moradi et al., 2013). For instance, the lack of ability of listeners to incorporate non-verbal context can contribute to miscommunication in aging, communication disorders, or under conditions like facial masking in healthcare settings (Rankin et al., 2009; Ujiie et al., 2015; Moradi et al., 2016; Nuber et al., 2018; Ramdani et al., 2022). In the case of animal signalers, visual displays or olfactory cues in combination with vocalizations may likewise clarify a signal relative to background noise or transform its meaning (e.g., Smith and Evans, 2008; Ronald et al., 2017, 2020; Caldart et al., 2022). These parallels in the use of context in human and animal communication mean that animal subjects can be valuable models for understanding the neural processes underlying how context modulates the perception of vocal signals.

Mice have a structured system of vocalizations that have been well-characterized and used as a model for studying communication disorders (Lahvis et al., 2011; Wöhr and Schwarting, 2013; Wöhr et al., 2015; Yao et al., 2023). Behaviorally, mice also show context-dependent production of vocalizations, and context-dependent responses to the vocal signals of other mice (e.g., Chabout et al., 2015; Hoier et al., 2016; Seagraves et al., 2016; Burke et al., 2018; Warren et al., 2018; Niemczura et al., 2020). For example, olfactory cues cause mice to respond differently to the same vocal signals in opposite-sex versus predatory contexts (Grimsley et al., 2013), while olfactory cues, hormonal state, and experience with foster fathers from different paternal strains help mice to demonstrate optimal preferences for vocalizations in a reproductive context (Asaba et al., 2014).

In an opposite-sex context, the ultrasonic vocalizations of male mice are widely thought to belong to a suite of reproductive behaviors, and are even described by some authors as courtship calls or songs (Arriaga et al., 2012; Egnor and Seagraves, 2016). In the initial stages of an interaction, males typically produce relatively high numbers of USVs as they investigate females nose-to-nose and anogenitally (Kikusui, 2013). Males produce USVs in response to pheromones in female urine, and produce a greater number of USVs when they have had prior experience with females (Haga-Yamanaka et al., 2014; Chabout et al., 2015). Males vocalize more when they have been previously “sexually primed” by experience with a female (Nyby et al., 1983; Roullet et al., 2011; Zala et al., 2020). On their side, females are attracted to playback of male USVs, and this attraction is influenced by female gonadal hormones and the presence of male pheromones (Asaba et al., 2014; Beck et al., 2020). During initial stages of interactions with males, females may produce audible squeaks (BBVs) when they are being vigorously contacted by males, and squeaks may be paired with physical aggression directed against males (Sugimoto et al., 2011; Finton et al., 2017). Higher numbers of squeaks in early stages of the interaction are associated with fewer male USVs and less male mounting in a later interaction stage (Finton et al., 2017). Although males and females produce USVs in multiple contexts, male USVs are different in structure and usage when males are paired with females or fresh female urine, and females prefer to approach calls based on these structural variations (Chabout et al., 2015).

In animal models including mice, one class of neural mechanism for representing communicative context is broadly projecting neuromodulatory systems, including the serotonergic and catecholaminergic (dopamine and norepinephrine) systems (Kiser et al., 2012; Riters, 2012; Barr and Woolley, 2018; Ghahramani et al., 2018, 2022; Alger et al., 2020). These systems function in part by integrating information on cues from a social partner with information on internal state. For example, catecholaminergic neurons across a range of vertebrate taxa show differences in activity related to the types of communicative signals animals are responding to Ghahramani et al. (2018) and Barr et al. (2021) and are also influenced by contextual factors such as sex and personality (Kelly and Goodson, 2015). Catecholaminergic activity in turn corresponds to the production of context-specific communication signals (Heimovics and Riters, 2008; Castelino and Schmidt, 2010; Riters, 2012; Ghahramani et al., 2022). Among other targets, catecholaminergic neurons provide feedback to sensory regions, modulating the ways that primary sensory systems process communication signals (e.g., Ikeda et al., 2015; Schofield and Hurley, 2018). Across animal systems, this process can calibrate sensory processing to different social contexts such as the presence or absence of a mating partner, and contribute to context-appropriate behavior (Castelino and Schmidt, 2010; Fast and McGann, 2017; Petersen and Hurley, 2017; Lee et al., 2018; Barr et al., 2021).

Like these catecholaminergic systems, the serotonergic dorsal raphe nucleus (DRN) is both responsive to social context and influences social behavior (Kiser et al., 2012; Schofield and Hurley, 2018). The serotonergic system has also been relatively well-studied in its influence on auditory processing. Specific subsets of DRN neurons project to multiple auditory regions, and have been associated with multiple types of social behaviors, including same-sex aggression, and mounting and olfactory investigation in an opposite-sex context (Klepper and Herbert, 1991; Niederkofler et al., 2016; Petersen et al., 2020). One of the auditory regions that is richly innervated by serotonergic inputs from specific regions of the DRN is the inferior colliculus (IC; Klepper and Herbert, 1991; Hurley and Thompson, 2001; Petersen et al., 2020). This midbrain region is a center for both ascending output to the auditory thalamus and descending input that regulates brainstem auditory nuclei (Ito et al., 2015; Chen et al., 2018; Balmer and Trussell, 2022; Lesicko and Geffen, 2022; Liu et al., 2023). Interactions between the IC and DRN are highly sensitive to social behaviors. The numbers of active DRN neurons in regions projecting to the IC in female mice correlate with male mounting behaviors as well as female olfactory investigation in an opposite-sex context (Petersen et al., 2020). Sex differences also emerge in the numbers of active neurons in midline groups of DRN neurons following opposite-sex interactions that are not present in the non-social context of restriction (Petersen et al., 2020). Within the IC, levels of serotonin in the males interacting with females inversely correlate with female squeaks, a signal of female defensive aggression that is often accompanied by kicks directed at males (Keesom and Hurley, 2016; Petersen et al., 2020). IC neurons themselves are responsive to the manipulation of serotonin, in both the numbers of neurons in the IC that are active following the playback of vocal signals, and the way that specific vocalizations are encoded by IC neurons (Davis et al., 2021; Gentile Polese et al., 2021). These findings suggest that serotonin plays a role in the context-dependent modulation of auditory processing.

**FIGURE 1:**
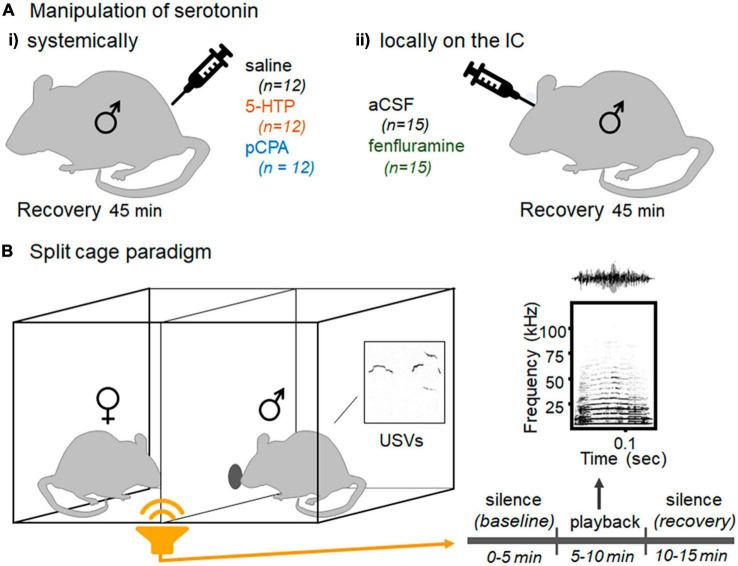
Experimental design **(Ai)** Mice were injected intraperitoneally with saline, 5-HTP solution, or pCPA solution. **(Aii)** A separate cohort of mice was infused locally in the IC with aCSF or fenfluramine solution. **(B)** The split cage paradigm, in which male and female mice are placed on opposite sides of a barrier with a small window for direct communication. Recorded female vocal signals, BBVs, are played through a speaker, preceded and followed by silent periods. Output measures include spontaneous USV production and non-vocal behaviors including grooming, digging in the bedding, and investigation at the window.

Despite the evidence of profound sensitivity to interactions with conspecifics in DRN-IC interactions, and the centrality of the IC in auditory pathways, it is unclear whether serotonin in the IC has any effect on conspecific-directed behavior. We tested this by manipulating serotonin in a novel behavioral assay that measures male vocal responses to female vocal signals: the split-cage assay schematically diagramed in Figure 1B (Hood et al., 2023). This assay reflects the interplay of two types of mouse vocalizations: ultrasonic vocalizations (USVs), and human-audible squeaks, also called broadband vocalizations (BBVs). In opposite-sex interactions, males produce most USVs, which have been called courtship “songs” in part because they attract females (Whitney et al., 1973; Hammerschmidt et al., 2009; Neunuebel et al., 2015; Sterling et al., 2023). Females are the main producers of BBVs, which in early phases of an interaction co-occur with kicks directed at males in response to vigorous male investigative contact, suggesting that they are part of a suite of defensive behaviors (Sugimoto et al., 2011; Lupanova and Egorova, 2015; Finton et al., 2017). In the split-cage assay, males and females are placed on opposite sides of a Plexiglas barrier with a small hole for limited contact, preventing vigorous male investigation of females (Hood et al., 2023). In this setup, males produce consistent and sustained ultrasonic vocalizations (USVs), which are a major output measure of the split-cage assay, but females do not produce BBVs, since they are not being directly contacted by males other than through the small hole in the Plexiglas barrier. This allows for the playback of BBVs though a speaker, which suppresses male USVs (Hood et al., 2023). This demonstrates that BBVs alone, in the absence of accompanying kicks, can alter male behavior. Male vocal behavior in the split-cage assay is context-dependent, in that it depends on having an awake female in the arena, and is altered by prior male social isolation (Hood et al., 2023). Within this assay in the current study, manipulation of serotonin levels both systemically and locally within the IC changed male vocal responses to BBV playback, suggesting a role for serotonin in contextual modulation of vocal behavior.

## Materials and methods

### Animals

A total of 77 male CBA/J mice were used as experimental subjects (Jackson Labs, Bar Harbor, ME), while 42 female mice were used in the split-cage assay to stimulate male social behavior (“stimulus” females). Mice were pair-housed with another experimental subject male except following surgery (see below), fed *ad libitum*, and kept on a 14:10 light:dark schedule, with the exception that males undergoing surgery were individually housed between surgery and behavioral testing to facilitate recovery. Males were ∼9 weeks of age and females were between 9 to 12 weeks of age at the time of behavioral testing. Experiments were carried out in accordance with the recommendations in the Guide for the Care and Use of Laboratory Animals of the National Institutes of Health. Protocols for all experiments were approved by the Bloomington Institutional Animal Care and Use Committee (protocols 18-025 and 21-020).

### Mouse courtship behavior and ultrasonic vocalizations

Male mice produce a higher number of vocalizations after having previous experience with females (Nyby et al., 1983; Roullet et al., 2011; Zala et al., 2020). Notably, this increase in male vocal response to females is observed after males have at least 5 min of interaction with females regardless of whether mounting or copulation occur during the interaction (Roullet et al., 2011; Zala et al., 2020). To give males experience interacting with a female before behavioral testing, each male was placed in the same undivided cage as a partner female, allowing direct interaction for 10 minute while any mounting was recorded, and males and females were rotated so that each individual was paired with multiple members of the opposite sex. This procedure was repeated daily until all males and females mounted or were mounted, respectively. Mounting was defined as a male in parallel with a female placing both arms over the female’s hindquarters. Mounting was usually accompanied by pelvic thrusts, but was usually not associated with intromission during the 10 min periods of observation. Females in these periods of observation never mounted. Mounting can occur across several contexts but in this opposite-sex interaction context where males are mounting females, we consider mounting to be a sexual behavior since it often precedes intromission (Nyby et al., 1983). Pairings continued until all males and females mounted or until males had undergone 2 days of opposite-sex experience (up to 12 total 10-min experiences). This opposite-sex experience was the only experience the experimental males had with females prior to playback experiments. Males were presented with novel stimulus females during playback experiments.

### Experimental design

Two main experiments were conducted to manipulate serotonin either systemically or locally within the inferior colliculus, as depicted in Figure 1A. The *systemic* study consisted of three groups of males (*n* = 12 each) administered either physiological saline (Covetrus, Portland ME), or 5-hydroxytryptophan (5-HTP; Sigma Aldrich, St Louis MO), or para-chlorophenylalanine methylester (pCPA; Sigma Aldrich, St Louis MO) dissolved in physiological saline. Although serotonin has poor penetration through the blood brain barrier (Bader, 2020; Jones et al., 2020), systemic injection of 5-HTP, a precursor to serotonin, results in an increase in neural serotonin levels (Birdsall, 1998), including a relatively rapid increase within the inferior colliculus after ∼30 min (Hall et al., 2010). In contrast, pCPA inhibits tryptophan hydroxylase, a synthetic enzyme for serotonin, resulting in the depletion of serotonin (Dailly et al., 2006; Ruhé et al., 2007).

The *local infusion* study consisted of two groups of males, one in which artificial cerebrospinal fluid (aCSF; Tocris Biosciences, Bristol, UK) was infused into the IC (*n* = 15), and one in which dexfenfluramine (Sigma Aldrich, St. Louis MO), a serotonin releaser and reuptake inhibitor (Rothman and Baumann, 2002), was dissolved in aCSF and infused (*n* = 19, but 4 were excluded as described below). When iontophoresed locally within the IC, fenfluramine alters neural responses to tones and frequency sweeps in the same way as serotonin (Hall and Hurley, 2007).

#### Drug administration for systemic study

Because pCPA must be administered over several days for maximal effect, all systemic treatment groups were given intraperitoneal injections daily for five consecutive days to equalize treatment among the groups, and to habituate mice to the injection procedure. Males in the saline group were given injections of saline on each of the 5 days, males in the 5-HTP group were given saline for the first 4 days and 5-HTP (50 mg/kg) on the fifth day. Because pCPA must be administered over multiple days to maximize its effect, males in the pCPA group were given injections of pCPA (200 mg/kg) on days 1–4 and saline on day 5. Day 5 was the day of behavioral testing, and injections were administered 45–60 min prior to behavioral testing.

#### Cannulation surgery and infusion

Guide cannulae were implanted above the IC of 41 mice using aseptic, stereotaxic surgery to allow for direct administration of drugs to the IC. These 41 mice were divided into 15 infused with aCSF, 19 infused with fenfluramine, and 7 in a pilot study as described below. Males undergoing implantation of a cannula guide (C23G-2.5/SPC 26GA 2.5 mm, Plastics One, Roanoke, VA) were placed under isoflurane anesthesia with a Somnosuite Small Animal Anesthesia System (Kent Scientific, Torrington, CT, USA). Anesthetic depth was assessed at least every 15 min with a tail or toe pinch to ensure a constant level of anesthesia. Before cannulae were implanted, mice were given 1 mg/kg of the analgesic Metacam via subcutaneous injection and their eyes were protected with a layer of Artificial Tears ophthalmic ointment (Henry Schein Animal Health, Melville, NY, USA). Hair in the surgical field was removed with depilatory cream and the field was sterilized with alternating washes of 70% EtOH and betadine. Mice were then placed in a stereotaxic apparatus (Stoelting, Wooddale, IL, USA), and the head was secured and leveled with the ear bars and nose clamp. An ∼1.5 cm incision was made along the midline of the skull, and the skin was reflected until both skull landmarks lambda and bregma were visible. The mouse’s nose was lowered until the landmarks lambda and bregma were approximately level (<0.2 mm difference between the two landmarks on the dorsal-ventral axis) and all subsequent distances were calculated relative to lambda. Two small holes, each ∼ 1 mm in diameter were drilled bilaterally over the IC at 1.1 mm posterior and ± 1.6 mm lateral to lambda. The Allen Mouse Brain Atlas was used as an anatomical reference to verify cannula placement (Allen Reference Atlas–Mouse Brain, 2011). A cannula guide was fitted with a dummy cannula (C235DC/SPC Dummy Double 0.2 mm, Plastics One, Roanoke, VA) and dust cap (303DC/1, Plastics One, Roanoke, VA) and was placed in the holes drilled above the IC. Dental cement was used to secure the cannula guide to the surface of the skull and a bone screw. After the dental cement fully dried, the incision was closed around the guide cannula with VetBond skin adhesive (3M, St. Paul MN), and the mice were returned to a cage to recover. Mice were housed individually and monitored daily after surgery and were given 1 mg/kg of Metacam the day after surgery, and on any subsequent day the mice lost weight. Behavior experiments occurred when the surgical incision was fully healed, and the mice showed no signs of pain or discomfort, between 5–8 days after surgery.

Forty-one mice were infused with either aCSF or fenfluramine in the IC via an internal cannula (C235I/SPC Internal Double 33GA 1 mm projection, Plastics One, Roanoke, VA) that had a depth of 3.5 mm from the surface of the skull. Seven mice were infused in a pilot with either 0.6 μL (*n* = 5) or 0.4 μL (*n* = 2) of solution to assess the spread of the infusate. Fifteen mice were infused with aCSF and 19 mice were infused with fenfluramine. Solutions were centrifuged through a 0.2-micron filter to remove potential pathogens before infusion. A 2.0 μL Hamilton Syringe (Hamilton Company, Reno NV) was attached to a sterile cannula with tubing and plastic connectors. Mice were anesthetized in the Somnosuite induction chamber before being placed on a heating pad. Anesthetized mice were given a subcutaneous injection of 1 mg/kg Metacam (Boehringer Ingelheim, Ridgefield, CT) and ophthalmic ointment over their eyes. The dust cap and dummy cannula were removed from the guide cannula and were replaced with the internal cannula connected to a Hamilton Syringe. A volume of 0.2 μL of either aCSF or 1 mg/mL of fenfluramine dissolved in aCSF was infused into each IC. This 0.2 μL volume was infused separately for each IC by depressing the Hamilton Syringe plunger over 1 min and then waiting another 4 min for the volume to diffuse. After infusion, cannulae were removed and replaced with dummy cannulae and dust caps. Mice were returned to their home cage to recover for at least 45 min before behavioral testing.

**FIGURE 2:**
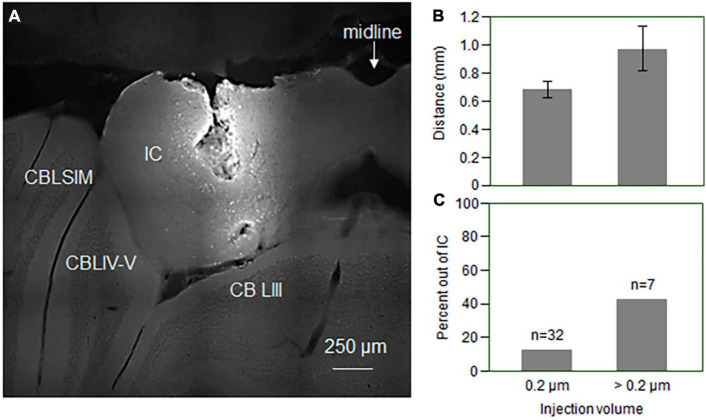
**(A)** Photomicrograph showing sites of injection into the IC. Cell bodies near the injection site have taken up neurobiotin. **(B)** Rostrocaudal extent of the injection sites in the IC is larger for larger injection volumes, but this effect is not significant. Error bars represent s.e.m. **(C)** Percentage of infusions with neurobiotin label appearing outside of the IC is higher for larger injection volumes. Volumes over 0.2 μm were either 0.4 μm (*n* = 2) or 0.6 μm (*n* = 5). Error bars represent s.e.m. IC, inferior colliculus; CBSIM, cerebellum, simple lobule; CBLIV-V, cerebellum, lobule IV-V; CBLIII, cerebellum, lobule 3.

For 39 of the 41 mice, infusion solutions were mixed with 1% neurobiotin (Vector Laboratories Inc., Newark, CA) to allow for visualization of the infusion site in the IC. These mice were perfused following behavioral testing after being euthanized by exposure to isoflurane fumes. Following a wash of Krebs-Henseleit saline, mice were perfused with 4% solution of paraformaldehyde. Brains were postfixed overnight, equilibrated in 30% sucrose, and sliced in 50 μm sections on a sliding microtome. Slices were exposed to fluorescent avidin (Vector Laboratories Inc.) to visualize the extent of the neurobiotin label, and imaged on a Delta Vision Elite microscope at 10x magnification using a GFP/FITC filter. An example section is shown in Figure 2A. Injection sites were compared for mice infused with volumes of 0.2, 0.4, and 0.6 μL of aCSF or fenfluramine solution. The rostrocaudal ranges of the neurobiotin volume were somewhat greater with larger injection volumes as shown in Figure 2B, but this difference was not significant (one-way ANOVA, *p* > 0.05). However, a higher proportion of brains with larger injection volumes showed neurobiotin outside of the IC (3/7 for 0.4 and 0.6 μL versus 4/32 in total for 0.2 μL; Figure 2C). We therefore only analyzed experiments using injection volumes of 0.2 μL, four of which, all in the fenfluramine group, ultimately showed labeling outside of the IC. These 4 individuals were excluded from analysis, leaving a total of 15 in the fenfluramine group.

### Dominance testing

Because social status has been reported to affect the numbers of USVs produced by male mice (Nyby et al., 1976), dominance in males housed together was determined using a tube dominance test. This test involved placing two males on opposite ends of a plastic tube with a diameter large enough for a single mouse to move through it freely but too narrow to allow the mice to pass over one another. Mice in this test are able to exit the tube only by turning around and leaving the side of the tube they entered or the mice can move forward through the tube and push the other male out of the tube. Both males were simultaneously released into the tube, and the first male to exit the tube was noted. If neither male left the tube after 1 min, the test was ended and marked as inconclusive. The tube and testing arena was cleaned with 70% EtOH between tests. Males that left the tube first were labeled as subordinate, and the males remaining in the tube were labeled as dominant, consistent with previous tube-dominance tests (Lindzey et al., 1961; Wang et al., 2014). Each male pair was tested twice daily between 9 and 11 AM for 2 days before recording. Males were only labeled as dominant or subordinate if they consistently performed as such on the two tube-dominance tests immediately before recording. Dominance testing was conducted in the systemic injection study but not in the infusion study, since mice were housed individually following cannula implantation in the latter.

### Playback experiment

Male behavior in response to playback of female BBVs was measured using a procedure described in Hood et al., 2023 (Figure 1B). Males were placed on one side of a standard mouse cage bisected by a plexiglass barrier with a small window (large enough for the tip of the nose of an adult mouse to fit through) for investigation, with a novel female social partner on the opposite side. The divided cage was contained in a sound-attenuation chamber (IAC Acoustics, Naperville, IL). Soiled bedding from the cage of the same female was additionally placed on the male side of the divided cage because this additionally stimulates USV production (Hood et al., 2023). The opening in the divider allowed for males to perform some investigation at the window but not the more aggressive physical contact that often results in female rejection behavior. This arrangement results in an absence of BBVs produced by females, so that no BBVs are produced by either of the mice in this assay. The BBVs played through the speaker are therefore the only BBVs that the mice hear (Hood et al., 2023).

Males were placed in the divided cage and were allowed to habituate to the cage until they began producing USVs, which was less than 3 min in all cases. Females were then placed on the other side of the divider, and the playback file was started. The playback file consists of 5 min of silence to establish a baseline of USV production, 5 min of BBV playback, and then another 5 min of silence to measure recovery of USV production (Figure 1B; described in Hood et al., 2023). There is large variation in BBV structure both within and between individual females (Finton et al., 2017). To control for the impact of BBV structure on behavior, an exemplar BBV was selected. The BBV playback was created taking the timing of BBVs produced by a female interacting with a male in a previous experiment for 1 min, replacing each BBV with the exemplar BBV, and then repeated 4 more times for 5 total minutes of playback (Hood et al., 2023). This playback file suppresses male USVs in the split-cage assay, but so do additional exemplars (Hood et al., 2023) or a mix of BBVs played in the same pattern (unpublished data). An Ultrasonic Dynamic Speaker (Vifa, range from 1 to 120 kHz) powered by an UltraSoundGate Player 116 (Avisoft Bioacoustics, Glienicke/Nordbahn, Germany) was used to play the BBV playback file. Both video and audio were recorded for playback trials. The video was recorded with a Canon VIXIA video camera placed above the divided cage (Q-See, Digital Peripheral Solutions Inc., Anaheim, CA) and a Q-See four-channel DVR PCI video capture card. Vocal behavior was recorded using a condenser microphone (CM16/CMPA, Avisoft Bioacoustics, Glienicke/Nordbahn, Germany) placed above the male side of the divided cage. The playback was played through an Ultrasonic Dynamic Speaker (Vifa/range from 1 to 120 kHz) powered by an UltraSoundGate Player (Avisoft Bioacoustics, Glienicke/Nordbahn, Germany). The intensity of the playback was calibrated as in Hood et al. (2023), based on the intensity of calls in a microphone close to a female producing a BBV.

### Behavioral analysis

#### Vocal behaviors

Vocalizations were analyzed in Avisoft SASLab Pro (Avisoft Bioacoustics, Glienicke/Nordbahn, Germany). Files were high-pass filtered at 40 kHz, and Avisoft’s whistle tracking function was used to count the number of USVs. In addition to total number of USVs, observers also counted USVs in a category with pronounced harmonics at ∼50 kHz and ∼100 kHz (“harmonic” USVs), which are enriched around the time of mounting (Hanson and Hurley, 2012). These are functionally and structurally distinct from “50 kHz” calls made by rats, in that mouse harmonic calls are produced around the time of mounting of males by females, and are interpreted as an indication of sexual behavior in opposite-sex interactions (Hanson and Hurley, 2012; Finton et al., 2017). We characterized the harmonic versus non-harmonic calls rather than more elaborate classification schemes for mouse USVs (e.g., Scattoni et al., 2008; Hanson and Hurley, 2012). This is because there is strong behavioral evidence for functional distinctions between harmonic and non-harmonic calls (Hanson and Hurley, 2012; Finton et al., 2017; Keesom et al., 2017; Matsumoto and Okanoya, 2018), while more elaborate classification schemes emphasize statistical differences in call structure, often without accompanying behavioral function.

#### Non-vocal behaviors

Four trained observers quantified non-vocal behaviors using Behavior Observation Research Interactive Software (BORIS v. 7.12.6; Friard and Gamba, 2016). Video recording failed for one male in the 5-HTP treated group and for five males in the fenfluramine group, leaving sample sizes in these groups of 11 and 10, respectively. Observers were considered trained when they had inter-observer reliability of at least 95% accuracy with a training data set that measured the same non-vocal behaviors scored in this experiment. The duration that each male spent digging and grooming and the duration that both males and females spent investigating the opening in the plexiglass divider were scored. Digging was scored when males moved the bedding with their forelegs. Grooming was scored when males manipulated their fur with their paws or tongue. Finally, investigation was scored when the male or female put their nose through or within one head length of the opening in the plexiglass divider.

### Statistical analysis

Statistical analyses were performed in SPSS. Outcomes of the systemic injection experiment and local infusion experiment were assessed with parallel statistical models. The normality and homogeneity of data distributions across individual behavioral trials were assessed with Shapiro-Wilks and Levene tests, respectively. Comparison of the control and drug treatments for behaviors during the initial baseline period of silence were assessed with independent samples Kruskal-Wallis tests.

**FIGURE 3:**
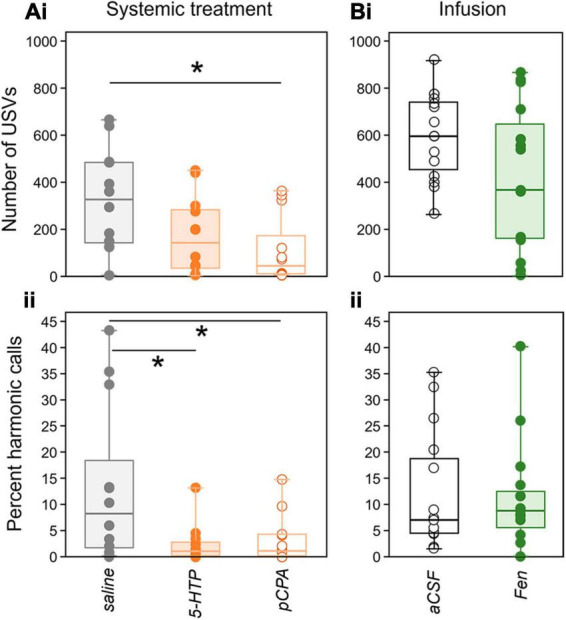
Effects of drugs on USV production in the baseline period (silence). **(A)** Systemic injection study. **(Ai)** Numbers of USVs are less in the pCPA treatment group than in the saline group, but do not differ between the saline and 5-HTP, or between the 5-HTP and pCPA treatment groups. **(Aii)** In contrast, the ratio of harmonic to non-harmonic calls is lower in both the 5-HTP and pCPA treatment groups compared to the saline group. **(Bi)** Numbers of USVs are not different in the aCSF and fenfluramine treatment groups, and **(Bii)** harmonic ratios also do not differ between these groups. Asterisks represent significant differences based on *post-hoc* tests; *p < 0.05.

For analysis of the playback trials, counts of vocal behaviors and durations of non-vocal behaviors were summed for each of the three 5-min time periods (baseline, playback, recovery). pCPA-treated mice showed low baseline calling behavior relative to controls (Figure 3Ai), and additionally lost significant weight relative to saline injections [0.56 ± 0.13 g for pCPA and 0.06 ± 0.19 g for saline, General Linear Model, *F*_(1_,_22)_ = 7.472, *p* = 0.012]. We therefore excluded pCPA-treated mice from analysis of behavioral responses to the playback of BBVs. Each behavior was modeled as a dependent variable using generalized linear mixed models with time period as a repeated measure and drug treatment as a between-subjects factor. A Poisson distribution was specified for USV counts, while a gamma distribution was used for the summed duration measurements for investigation, digging, and grooming. Since these assumptions require non-zero values, all call numbers and duration values for non-vocal behaviors were transformed by adding a value of one. Dominance status had no main effect or interaction with drug treatment or time period, so was excluded from final models. Tukey’s Least Significant Difference was used for *post-hoc* testing, specifying a link to the underlying data distribution. A general linear model was used to assess differences in proportional decline of USVs between the baseline and playback periods, with drug treatment as a between-subjects factor. Spearman correlations were used to compare male versus female investigation behavior across behavioral trials. The Benjamini-Hochberg procedure was used to control false discovery rates for generalized linear mixed models across multiple behaviors, for the comparisons in proportional declines in control versus drug treatment across the different experiments, and for Spearman correlations between male and female investigation behavior across drug treatments in different groups of mice. All Benjamini-Hochberg adjustments used a Q value of 0.05.

## Results

### Baseline differences in vocalizing occur for systemic but not local manipulation of serotonin

In the systemic study, numbers of USVs in the baseline “silent” time period were different among drug treatment groups [Kruskal-Wallis test, *H*(2) = 6.98, *p* = 0.03]. Figure 3 depicts measures of USVs in the baseline time period in all drug treatment groups in the systemic injection (left) and local infusion (right) studies. The top row (3Ai and 3Bi) shows USV numbers, while the bottom row (3Bi and 3Bii) depicts the per cent of harmonic calls. Although the group treated with pCPA, the serotonin depleter, produced a lower number of baseline USVs than the saline group (*p* = 0.009), the saline and 5-HTP groups, and the pCPA and 5-HTP groups, were not significantly different (Figure 3Ai: pCPA vs. 5-HTP: *p* = 0.323; saline vs. 5-HTP: *p* = 0.104). The percentage of USVs in the “harmonic” category, which are elevated around the time of mounting during direct opposite-sex interactions (Hanson and Hurley, 2012), were also calculated (Figure 3Aii). The percentage of harmonic calls that males produced in the baseline time period was lower than the saline treatment in both the 5-HTP treatment group, and in the pCPA treatment group [Figure 3Aii; 1.ii; Kruskal-Wallis test, *H*(2) = 6.07, *p* = 0.048; saline vs. 5-HTP: *p* = 0.028; saline vs. pCPA: *p* = 0.04]. In contrast, there were no differences between the aCSF and fenfluramine treatment groups in either the numbers of USVs [Figure 3Bi; one-way ANOVA, *F*(1,28) = 3.61, *p* = 0.068] or the percentage of harmonic calls [Figure 3Bii; *H*(1) < 0.001, *p* = 0.983] produced in the baseline time period. Baseline differences in vocal behavior therefore depended on both the type of call and the type of drug treatment.

### Playback and drug treatment affect USVs

**FIGURE 4:**
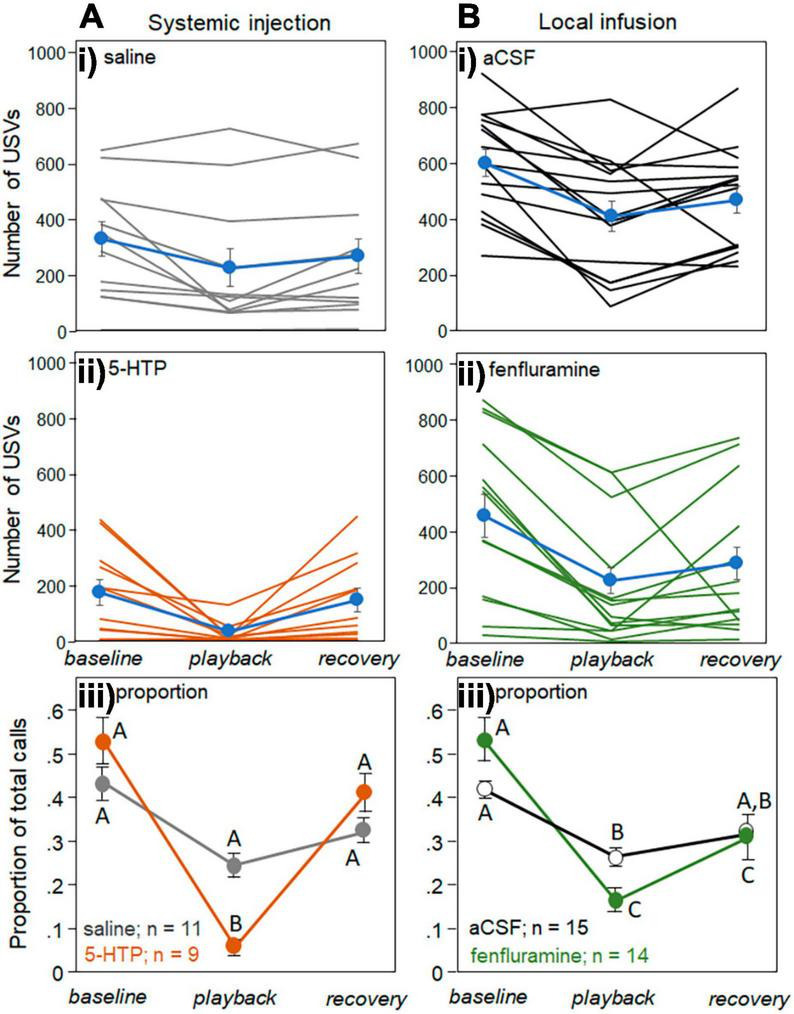
Effects of drugs on USV production across the baseline, playback, and recovery time periods. **(A)** Systemic injection study and **(B)** local infusion study. Numbers of USVs in each trial between a male and novel female for the control treatments of saline and aCSF **(Ai,Bi)**, and for the drug treatments of 5-HTP and fenfluramine **(Aii,Bii)**. Blue lines represent averages in each condition. **(Aiii)** and **(Biii)** show average proportions of USVs relative to the total number of USVs across individual trials for the systemic injection experiment **(Aiii)** and the local infusion experiment **(Biii)**. Proportions across the three time bins add to 1. Sample sizes represent numbers of interactions in which the number of USVs in the baseline period were above 10. Error bars represent the s.e.m.

Playback of female BBVs decreased the USVs produced by males, as previously reported in Hood et al. (2023). For the systemic treatment, the pCPA group was excluded, since half of this group had baseline USV numbers of 10 or less, making it difficult to assess decreases in calls. Figure 4 shows the raw numbers of USVs produced in every behavioral trial of the systemic injection study following the injection of saline (Figure 4Ai, gray lines) and 5-HTP (Figure 4Aii, orange lines). USV numbers for each individual behavioral trial during the different time periods (the 5 min baseline, 5 min playback, and 5 min recovery) are connected. Average values for each of the drug treatments (blue symbols and lines) generally show a decline in USV number during playback, and a partial increase during the recovery period. Consistent with previous findings (Hood et al., 2023), there was substantial variation in the calling rate across individuals. To account for differences in calling baseline, Figure 4Aiii shows the USV numbers in both the saline and 5-HTP treatment groups as proportions of the total number of USVs in the behavioral trial, so that proportions across the three time periods sum to 1. Figure 4B depicts parallel data for the local infusion study, with raw USV numbers for the aCSF treatment group (Figure 4Bi), raw numbers for the fenfluramine treatment group (Figure 4Bii), and proportional values for each group (Figure 4Biii).

Generalized mixed models were used to assess the effects of time period as a repeated measure and drug treatment as a between-subjects factor, in both the systemic and local infusion experiments (Table 1). The time period and drug treatment each had significant effects in both the systemic injection and local infusion studies, such that USVs decreased during playback and were lower overall in the drug treatments. For the systemic study, there was a further significant interaction between the time period and drug treatment. The letters in Figures 4Aiii, Biii represent the results of *post-hoc* tests on the raw numbers of USVs.

**TABLE 1:** Generalized mixed model for the effects of drug treatment and time period on USVs.

Systemic injection					Local infusion				
Number of USVs	*F*	df1	df2	Sig.	Number of USVs	*F*	df1	df2	Sig.
Treatment	6.529	1	66	0.013	Treatment	6.95	1	84	0.01
Bin	6.214	2	66	0.003	Bin	20.693	2	84	<0.001
Treatment × bin	4.111	2	66	0.021	Treatment × bin	2.998	2	84	0.055

**FIGURE 5:**
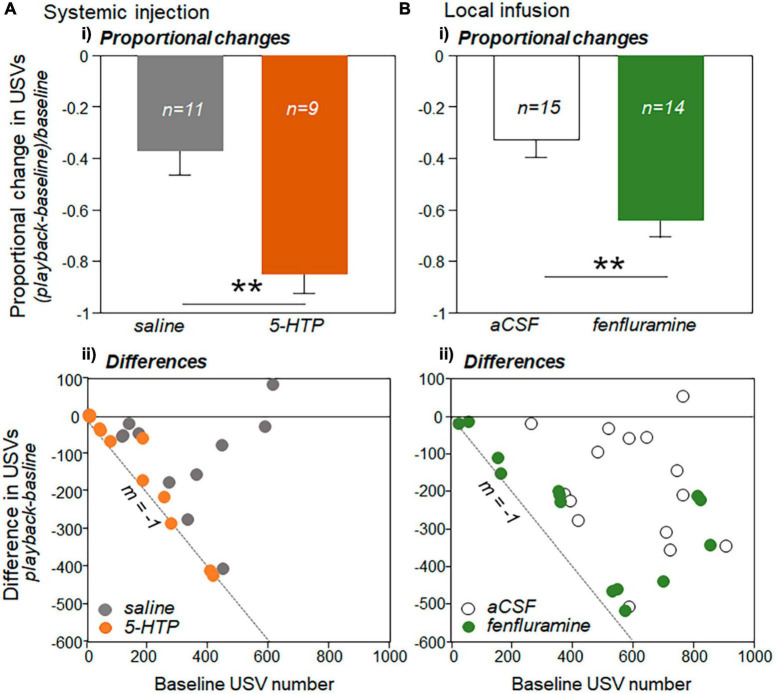
Changes in USV production during playback relative to baseline for **(A)** the systemic injection experiment and **(B)** local infusion experiment. **(Ai)** Proportional changes in USV number during playback (playback-baseline)/baseline. Proportional decrease is significantly larger in the 5-HTP injection group than in the saline group. **(Bi)** The proportional decrease is also significantly larger for the fenfluramine group than the aCSF group. Sample sizes represent numbers of interactions in which the number of USVs in the baseline period were above 10. Differences in USV number between the baseline and playback period (Y-axis: playback – baseline) versus the baseline USV number (x-axis) are plotted for the systemic injection experiment **(Aii)** and the local infusion experiment **(Bii)**. Dashed lines have a slope of –1, representing a total decrease in USVs during the playback. Error bars represent s.e.m. Asterisks represent significant differences based on *post-hoc* tests; **p < 0.01.

Figures 4Aiii, Biii suggest that proportional decreases in USVs were greater in each of the drug treatments (5-HTP injection and fenfluramine infusion) than in their respective controls (saline and aCSF). To directly assess this possibility, the proportional change in USVs in the playback relative to the baseline period was calculated for each male as the decrease in USVs normalized by the baseline call number: (playback – baseline)/baseline (Figures 5Ai, Bi). For this analysis, one male in the saline group, three in the 5-HTP group, and one in the fenfluramine group were excluded due to baseline USV numbers that were under 10 in total. For both the systemic injection and local infusion experiments, the drug treatment showed a significantly greater proportional decrease in USVs than the control treatment, with proportional decreases in USVs approximately doubling in the drug treatments relative to controls [independent Kruskal-Wallis tests for saline vs. 5-HTP, *H*(1) = 10.43, *p* = 0.001; for aCSF vs. fenfluramine, *H*(1) = 8.05, *p* = 0.004].

To assess whether the larger proportional decreases in USVs could simply be a result of somewhat lower baseline numbers in the drug treatment groups (Figures 3Ai, Bi), we examined the difference in numbers of USVs versus the baseline USV number for the control and drug conditions for both the systemic injection and local infusion studies. This comparison is shown for the systemic injection versus local infusion studies in scatterplots in Figures 5Aii, Bii. The black dashed lines in Figures 5Aii, Bii have a slope of −1, representing a 100% decrease in calls in the playback relative to the baseline. Although there are more males with low baseline numbers of USVs close to the line with *m* = −1 for both the 5-HTP (Figure 5Aii, orange circles) and fenfluramine (Figure 5Bii, green circles) treatments than their respective controls (gray and white circles), the trials with higher numbers of baseline USVs were also generally closer to *m* = −1 for the drug treatments than their respective controls. To quantify this distribution, the residuals of differences in USV numbers from the line with *m* = −1 were measured for each data point and compared between the drug and control treatments. The residuals were greater for each of the respective control treatments than for the drug treatments [independent samples Kruskal-Wallis tests: for systemic injection experiment, *H*(1) = 9.38, *p* = 0.002; for the local infusion experiment, *H*(1) = 9.30, *p* = 0.002]. This demonstrates trials in the drug treatments were closer to the line with a slope of *m* = −1. This finding suggests that the larger proportional decreases in the drug treatment were not exclusively due to lower rates of calling, but represent greater responsiveness to BBV playback regardless of calling rate.

### Non-vocal behaviors

**FIGURE 6:**
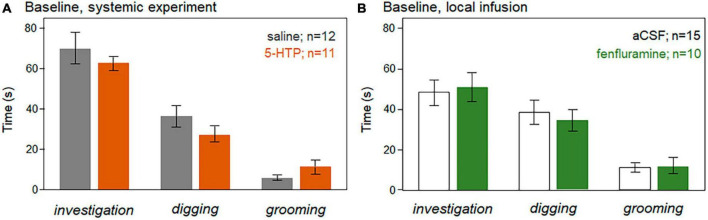
Average durations of the non-vocal behaviors of investigation of the window, digging, and grooming during the baseline time period in the systemic injection study **(A)** and the local infusion study **(B)**. There were no significant differences between the control treatments (saline, aCSF) versus the drug treatments (5-HTP, fenfluramine) for any behavior. Error bars represent s.e.m.

The summed durations of investigation of the barrier window, digging, and grooming were measured. Baseline values of these behaviors were not significantly different in the control versus drug treatments for either the systemic injection or local infusion studies (independent Kruskal-Wallis tests, *p* > 0.05), as depicted in Figure 6A for the systemic injection study and Figure 6B for the local infusion study.

**FIGURE 7:**
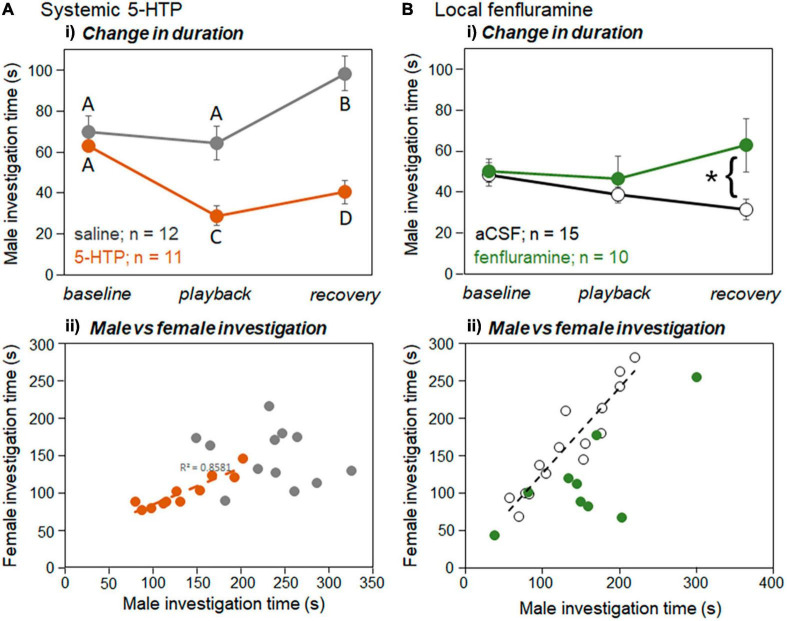
Effects of playback and drugs on investigation behavior for **(A)** systemic injections and **(B)** local infusions in the IC. **(Ai)** Mean durations of investigation of the window by males across baseline, playback, and recovery time periods for saline injections (gray symbols) versus 5-HTP injections (orange symbols). **(Bi)** Mean durations of investigation of the window by males across baseline, playback, and recovery time periods for aCSF injections (open black symbols) versus fenfluramine injections (green symbols). **(Aii)** Duration of male versus female investigation times across trials for injections of saline (gray circles) versus injections of 5-HTP (orange circles). Dashed line indicates significant linear regression between male and female durations in the 5-HTP treatment. **(Bii)** Duration of male versus female investigation times across trials for infusions of aCSF (open circles) versus injections of fenfluramine (green circles). Dashed line indicates significant linear regression between male and female durations in the aCSF treatment (black line). Error bars represent s.e.m. Asterisks represent significant differences based on *post-hoc* tests; *p < 0.05.

Investigation behavior significantly changed over time periods and across drug treatments, with some opposing effects for systemic 5-HTP and local fenfluramine (Table 2). Figure 7 illustrates the effects of these different drug treatments. For the systemic injection study, there was a significant effect of drug treatment, with investigation lower in 5-HTP than saline (Figure 7Ai; Table 2). Across saline and 5-HTP treatments, investigation time was lower during playback than in the baseline or recovery. Additionally, the drug treatment significantly interacted with the time period. Following the injection of saline (Figure 7Ai, gray symbols), investigation time did not change during the playback but increased in the recovery period. In contrast, following the injection of 5-HTP (Figure 7Ai, orange symbols), investigation time declined during the playback period and was also low during the recovery. For the local infusion study, there were no effects of drug treatment or time period, so that investigation in neither the aCSF nor the fenfluramine treatment changed over time period (Table 2). However, there was interaction between the two, with the fenfluramine-treated males showing increased inspection time relative to aCSF-treated males (Figure 7Bi). It is also interesting to note the differing patterns of change in investigation for the control treatments of the two experiments, with investigation increasing in the recovery period in saline but remaining stable in aCSF.

**TABLE 2:** Generalized mixed model for the effects of drug treatment and time period on investigation, digging, and grooming.

Systemic injection					Local infusion				
Investigation(s)	*F*	df1	df2	Sig.	Investigation(s)	*F*	df1	df2	Sig.
Bin	14.374	2	63	<0.001	Treatment	2.264	1	69	0.137
Treatment	22.11	1	63	<0.001	Bin	0.992	2	69	0.376
Treatment × bin	11.693	2	63	<0.001	Treatment × bin	4.028	2	69	0.022
**Digging(s)**					**Digging(s)**				
Treatment	1.557	1	63	0.217	Treatment	0.353	1	69	0.554
Bin	16.914	2	63	<0.001	Bin	11.448	2	69	<0.001
Treatment × bin	6.086	2	63	0.004	Treatment × bin	0.038	2	69	0.962
**Grooming(s)**					**Grooming(s)**				
Treatment	5.15	1	63	0.027	Treatment	0.831	1	69	0.365
Bin	24.727	2	63	<0.001	Bin	51.677	2	69	<0.001
Treatment × bin	0.742	2	63	0.48	Treatment × bin	1.235	2	69	0.297

We recently showed that male investigation time and female investigation time in the split-cage assay were correlated across interactions regardless of the time period, potentially as a result of direct male-female interaction at the window (Hood et al., 2023). In the current study both the injection of 5-HTP and the infusion of fenfluramine affected this correlation, but in different ways. For the systemic injection study, there was no correlation in the saline treatment between male and female investigation time (Figure 7Aii, gray symbols; Spearman correlation, *r* = −0.154, *p* = 0.633). In the 5-HTP treatment, although the amount of investigation was lower than in 5-HTP by both males and females, there was a strong correlation between male and female investigation (Figure 7Aii, orange symbols; Spearman correlation, *r* = 0.855, *p* < 0.001). For the local infusion study, the correlations were reversed, with the aCSF but not fenfluramine treatment showing a significant correlation between male and female investigation time (Figure 7Bii; Spearman correlations, aCSF: *r* = 0.961, *p* < 0.001; fenfluramine: *r* = 0.627, *p* = 0.035, not significant after Benjamini-Hochberg correction for multiple comparisons). Supplementary Table 1 shows uncorrected values for Pearson’s correlations for male and female investigation behaviors across the baseline, playback, and recovery time periods. In general, uncorrected correlations had *p*-values of less than 0.05 for treatments except for the saline treatment, and not for the baseline period in the 5-HTP treatment (Supplementary Table 1). Female investigation time was also not correlated with either male USV number or percent harmonic calls in any of the drug treatments in either experiment (Spearman correlation, *p* > 0.05 for all tests).

**FIGURE 8:**
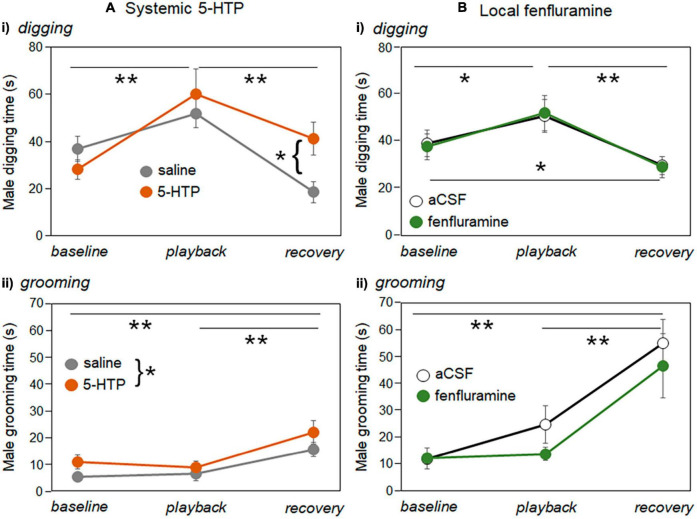
Changes in the durations of digging and grooming behavior production **(A)** the systemic injection experiment and **(B)** local infusion experiment. **(Ai)** Mean durations of digging by males across baseline, playback, and recovery time periods for saline injections (gray symbols) versus 5-HTP injections (orange symbols). **(Bi)** Mean durations of digging by males across baseline, playback, and recovery time periods for aCSF injections (open black symbols) versus fenfluramine injections (green symbols). **(Aii)** Mean durations of grooming by males across baseline, playback, and recovery time periods for saline injections (gray symbols) versus 5-HTP injections (orange symbols). **(Bi)** Mean durations of grooming by males across baseline, playback, and recovery time periods for aCSF injections (open black symbols) versus fenfluramine injections (green symbols). Asterisks represent significant differences based on *post-hoc* tests; *p < 0.05, **p < 0.01. Error bars represent s.e.m.

For digging and grooming, there were mixed effects of drug treatments on the duration of these behaviors (Table 2). Figure 8 illustrates the duration of digging and grooming behaviors in the systemic injections study (Figure 8A) and the local infusion study (Figure 8B). Digging and grooming showed generally similar trajectories across time period in all treatments, with digging increasing during playback and declining in the recovery (Figures 8Ai, Bi) and grooming not changing during the playback but increasing during the recovery (Figures 8Aii, Bii). These patterns are reflected in the significance of the factor of time period in all generalized mixed models (Table 2). In the systemic injection study, there was an additional interaction between time period and drug treatment for digging, driven by a higher level of digging in 5-HTP treated males in the recovery time period (Figure 8Ai), and a main effect of drug treatment on grooming, with higher levels of grooming in males injected with 5-HTP (Figure 8Aii). Supplementary Table 2 contains the data used in these analyses.

## Discussion

Models of neuromodulatory feedback to sensory systems suggest that brain regions like the dorsal raphe nucleus integrate information on multiple aspects of a given social interaction (Kiser et al., 2012; Petersen and Hurley, 2017; Lee et al., 2018; Hurley, 2019; Barr et al., 2021). These regions project to sensory areas, among other targets, modulating how sensory neurons respond to conspecific cues and ultimately contributing to contextually appropriate behavioral responses. Across a range of animal models, neuromodulatory systems like the dopaminergic, serotonergic, and noradrenergic systems modify or gate sensory responses to communication signals (Hurley et al., 2004; Jacob and Nienborg, 2018; Sizemore et al., 2020).

We tested this hypothesis by assessing whether the manipulation of serotonin affects the conspecific-directed and non-social behaviors of male mice to female vocal signals. The type of female vocal signal we used as a stimulus was a BBV, or squeak. This is a human-audible call that mice of all sexes make in distress (Ruat et al., 2022). In opposite-sex interaction, females make BBVs and a minority of USVs, while males produce the majority of USVs (Whitney et al., 1973; Lupanova and Egorova, 2015; Neunuebel et al., 2015; Sterling et al., 2023). BBVs are produced in part during the appetitive stages of opposite-sex interactions in response to close male investigation, while females are concurrently kicking or lunging at males, but also when males are mounting females (Sugimoto et al., 2011; Lupanova and Egorova, 2015; Finton et al., 2017). We assessed the male response to BBVs in a newly described behavioral paradigm that replicates a rejective BBV context, the split-cage assay (Hood et al., 2023). In this assay, males and females are separated by a barrier. The presence of awake females and the olfactory stimulus of soiled bedding creates a context in which males are highly motivated to produce sustained USVs over time and investigate the small window in the barrier. Because males are unable to directly investigate females, females do not produce BBVs, providing the opportunity to play back recorded BBVs in a controlled manner. Males suppress their USV production during periods of BBV playback in this assay.

Using the split-cage assay, we pharmacologically manipulated serotonin locally within a central midbrain auditory processing region (the IC), and more broadly via systemic injection. We found that serotonin did alter the behavior of male mice in response to the playback of female BBVs. Below we describe the main conclusions and caveats of our study, fit our findings into the context of the sensory feedback model of neuromodulation, and discuss the relevance of our findings and model for studies of human context and communication.

### Serotonin makes males more responsive to female signals

We pharmacologically manipulated serotonin in our study through two distinct strategies. We locally increased serotonin levels within the IC by using bilateral cannulae to infuse fenfluramine, a drug that blocks serotonin reuptake and causes direct serotonin release (Rothman and Baumann, 2002). We compared this manipulation to a separate set of experiments in which serotonin was more broadly manipulated with systemic injection of 5-HTP, a serotonin precursor (Birdsall, 1998). Although neither of these two treatments significantly decreased the overall numbers of USVs in the baseline period of silence, both caused increased proportional and absolute responses of male mice to the playback of female rejection calls (BBVs). During BBV playback, males decreased the production of USVs more when exposed to 5-HTP and fenfluramine than in the respective controls of saline and aCSF. The greater suppression of USVs did not depend on the initial calling rate of particular males, but was seen across the range of baseline calling rates, which varied widely among behavioral trials.

Fenfluramine application in the IC mimics the effects of serotonin on neural responses to sound (Hall and Hurley, 2007), consistent with its effects occurring through the elevation of serotonin. An elevation in serotonin levels induced by fenfluramine could alter neural activity through a range of receptor types expressed by the IC neurons themselves. Serotonergic receptors in 5 different families are expressed in the IC (Hurley and Sullivan, 2012). Of these, iontophoretic manipulation of the 5-HT1A and 5-HT1B, 5-HT2, and 5-HT3 receptors all modulate the responses of IC neurons to sound (Hurley, 2006, 2007; Hurley et al., 2008; Bohorquez and Hurley, 2009; Ramsey et al., 2010). Activation of different receptor types can have suppressive or facilitatory effects on auditory responses in the IC, and can also alter the temporal structure of responses. The application of serotonin itself, or agonism of the 5-HT1A receptor, have further been shown to modulate responses to USVs or BBVs (Hurley and Pollak, 2005; Gentile Polese et al., 2021). These findings all demonstrate that serotonin alters the way that that sounds, including BBVs, are encoded by auditory neurons. The IC is also part of a complex of midbrain regions that can evoke defensive behaviors like running and freezing, and also influence autonomic responses (Brandão et al., 1993). Behaviors indicative of aversion evoked by stimulation of the IC are modulated by microinjection of serotonin reuptake inhibitors or serotonin receptor agonists or antagonists (Brandão et al., 1993; Melo and Brandão, 1995). Our infusions of fenfluramine into the IC could therefore be acting by changing auditory responses to BBVs, or by influencing an important substrate of defensive behaviors.

Although both local infusion of fenfluramine and systemic injection of 5-HTP both increased the responsiveness to female BBVs, the two types of drug manipulations had divergent effects in other ways, with the systemic manipulation of serotonin generally affecting a wider range of behaviors. Systemic 5-HTP injection, although it did not significantly decrease USVs in the baseline time period, dramatically decreased the ratio of harmonic to non-harmonic USVs. Since harmonic USVs often occur around mounting behavior (Hanson and Hurley, 2012; Matsumoto and Okanoya, 2018), this finding raises the possibility of a change in sexual motivation. In contrast, infusion of fenfluramine into the IC did not have an effect on the ratio of harmonic calls. 5-HTP injection also had a strong effect on investigation behavior. Again, this effect was not seen in the baseline period, but the males in the 5-HTP group exhibited less investigation of the barrier window during and after the playback relative to the saline-treated males. The correlation between male and female time at the window across behavioral trials, a possible indication of mutual investigation, was also observed in the 5-HTP group but not the saline group. Fenfluramine had the opposite effects on investigation, increasing investigation during the recovery period and reducing correlation of male and female investigation relative to aCSF. In contrast to USV production and investigation behavior, digging and grooming showed little effect of either type of serotonergic manipulation. All together, these results suggest that serotonin increases male responsiveness to female vocal signals in the split-cage assay, and has stronger effects on potentially conspecific-directed (vocalizing, investigating) than on non-social (digging, grooming) behaviors, in line with an important role for serotonin in regulating social behavior.

A caveat to this conclusion is that because males and females are separated in the split-cage assay, males were unable to demonstrate sexual behaviors such as anogenital investigation and mounting. Male behaviors in the split-cage assay parallel male behaviors in direct interactions with females in that males produce more USVs in the presence of female soiled bedding, and males reduce USVs in response to BBVs (Hood et al., 2023). Thus, although we think it most plausible that male behavior toward females in this assay represents sexual interest and courtship, it is possible that it is representative of a more general social response. Additionally, we did not measure the estrous phases of stimulus females. We and other authors have found that female estrous phase does not affect the numbers of calls produced by males, or the relative proportions of different syllable types (Hanson and Hurley, 2012; Kim et al., 2016). We have found that estrous phases of females affects the dominant frequency, bandwidth, and duration of male syllables, but we did not measure these aspects of the calls in this study.

Differences between the effects of systemically injected 5-HTP and locally infused fenfluramine could arise from several causes. One of these is the site of action, with the systemic treatment affecting serotonin levels across a wider range of brain regions that could potentially affect vocal behavior. For example, a group of neurons in the caudolateral periaqueductal gray triggers USV production (Tschida et al., 2019). These neurons act as a gate integrating behaviorally relevant inputs through a range of parallel neural circuits (Michael et al., 2020; Chen et al., 2021; Xiao et al., 2023), although whether the components of these circuits are sensitive to serotonin is unknown. Although the PAG is physically close to the IC, we believe that the local injections of fenfluramine in the IC are unlikely to have affected the USV-gating neurons directly. This is partly because of our care in excluding cases in which tracer was found outside of the IC, but also because any direct effects of fenfluramine on PAG neurons would likely affect baseline USV rates. Fenfluramine had no significant effect on non-harmonic or harmonic baseline USV rates, but significantly decreased USVs during the playback of BBVs. 5-HTP, causing a strong decrease in the harmonic ratio, could have been affecting sites in the PAG or other regions that regulate harmonic USV production.

An additional reason for differences between the systemic and local drug manipulations is the different pharmacological profile of each of the drugs. As a precursor to serotonin, 5-HTP acts to increase serotonin synthesis, while fenfluramine blocks serotonin reuptake and causes direct serotonin release (Birdsall, 1998; Rothman and Baumann, 2002). In the IC, localized fenfluramine mimics the effects of serotonin (Hall and Hurley, 2007), while systemically administered 5-HTP increases directly measured serotonin levels (Hall et al., 2010), suggesting that both drugs increase serotonin availability. However, the two drugs could also have different “off-target” effects. For example, 5-HTP may increase central levels of melatonin, which is synthesized from serotonin (Birdsall, 1998), while fenfluramine could affect opioid receptors as well as increase serotonin levels (Samanta, 2022). Both opioid and melatonin receptors are expressed by IC neurons (Tongjaroenbuangam et al., 2006; Lacoste et al., 2015), providing a substrate for such “off-target” effects. The modes of administration- 5 days of injections in the systemic study and surgical implantation of cannulae in the infusion study- could themselves cause different types of plasticity in the stress-sensitive serotonergic system (Holmes, 2008; Paul et al., 2014; Baratta et al., 2023). Finally, mice in the local infusion study were individually housed following surgery before the behavioral trials, while mice in the systemic injection study were socially housed. Periods of isolation may increase the vocal behavior of male mice (e.g., Chabout et al., 2012; Keesom et al., 2017), so this difference could have accounted for the elevated USV numbers in the baseline time period for the saline-treated males in the systemic injection study versus the aCSF-treated males in the local infusion study (Figures 3, 4). These methodological differences could also have accounted for the different patterns of change in male investigation over the baseline, playback, and recovery time periods. These potential distinctions make the similarities in the effects of the drugs on male responses to female calls even more striking.

### Sensory feedback model

**FIGURE 9:**
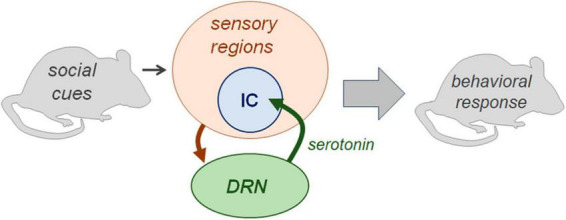
Diagram illustrating aspects the sensory feedback model examined in the current article. Neurons of the dorsal raphe nucleus integrate information on social cues and project to brain regions including the IC. This interaction is capable of influencing the behavioral responses to social cues. Not depicted are additional sources of input on context to the DRN, additional targets of DRN neurons, and additional brain regions involved in the representation of behavioral context.

The sensory feedback model as it applies to serotonergic effects in the IC (illustrated in Figure 9) has support in anatomical and physiological lines of evidence. DRN neurons in regions projecting to the IC create a dense network of axons and release sites, and show immediate early gene activity that is correlated with social behaviors (Klepper and Herbert, 1991; Hurley and Thompson, 2001; Petersen et al., 2020). In the IC itself, we have previously measured serotonin levels in the of males in opposite-sex interactions using the technique of carbon fiber voltammetry (Keesom and Hurley, 2016). In the IC of male mice, serotonin increases during interaction with a female, and the amplitude of the increase across individuals correlates inversely with female vocal signals (squeaks/BBVs; Keesom and Hurley, 2016). In the same study, the trajectory of increases in serotonin was not correlated with the production of harmonic USVs (made around mounting) relative to non-harmonic USVs. However, it is important to note that, even when serotonergic activity correlates with vocal behaviors, this does not necessarily imply a causal relationship. The DRN receives inputs from a wide range of brain regions (Pollak Dorocic et al., 2014; Weissbourd et al., 2014), so could release serotonin within the IC in response to non-auditory social cues. Moreover, highly localized applications of serotonergic drugs strongly affect the responses of IC neurons, including response to BBVs, in ways that can depend on cell type and the type of serotonin receptors that are activated (Hurley and Sullivan, 2012; Davis et al., 2021; Gentile Polese et al., 2021). The current study demonstrates that manipulation of serotonin within the IC, as well as more broadly, influences the behavioral response to vocalizations, providing key support for the feedback model.

This description of the interaction between social behavior, sensory systems, and the serotonergic system leaves an important functional question unaddressed: why should males suppress their courtship/communication calls more in response to female rejective signals when serotonin levels are high? High chronic levels of serotonin are stereotypically associated with reduced sexual behavior, although this broad generalization might not apply equally to all brain areas or serotonin receptor types (Hull and Dominguez, 2007; Pfaus, 2009; Angoa-Pérez and Kuhn, 2015). However, the enhanced suppression of USVs, often thought of as courtship vocalizations of male mice (Arriaga et al., 2012; Egnor and Seagraves, 2016), is consistent with a suppressive effect of serotonin on reproductive behavior. The stronger effects of serotonergic manipulation on harmonic USVs, which have been closely associated with sexual behavior, are consistent with this possibility. Multiple studies of sensory processing in animal models have suggested that serotonin contributes to a “shut up and listen” effect, in which self-produced communication signals are temporarily silenced in order to attend to the signals of a social partner (Deemyad et al., 2013; Jacob and Nienborg, 2018). USV suppression in the split-cage assay is consistent with this model, particularly since USVs are suppressed on a relatively rapid time scale of hundreds of milliseconds (Hood et al., 2023). Suppression of courtship could also benefit males by preventing injury, since BBVs are typically produced in association with female kicks or lunges (Sugimoto et al., 2011; Lupanova and Egorova, 2015; Finton et al., 2017). Courtship suppression could further assist in pacing courtship interactions; in rats, females that experience paced mating interactions have larger litter sizes (Coopersmith and Erskine, 1994).

### Relevance for studies of human communication

Some aspects of the experimental system and approaches we have described could be relevant for research on the mechanisms of emotionally salient vocal communication. Sounds with positive or negative qualities (valences) are perceived and processed differently than neutral sounds in brain regions like the amygdala, and multimodal cues such as facial expressions can influence the process of emotional judgment (Rankin et al., 2009; Skipper et al., 2009; Kumar et al., 2012; Gerdes et al., 2014; Husain et al., 2014; Buono et al., 2021). Age, hearing loss, and social isolation/loneliness can alter the evaluation of the emotional value of sounds, generally dampening the distinctions between neutral and pleasant or unpleasant sounds (Picou, 2016; Picou and Buono, 2018; Zinchenko et al., 2018; Christensen et al., 2019). These changes may in some cases be due to the loss of access to specific frequency channels (Buono et al., 2021), but neural processing of valenced sounds is also altered (Husain et al., 2014), suggesting an additional central component. Furthermore, the complex interactions among aging, hearing loss, and social isolation are associated with increased disorders of mood and cognition (Fortunato et al., 2016; Humes and Young, 2016; Mamo et al., 2017; Shukla et al., 2020; Brewster et al., 2021; Zhao et al., 2023).

Animal models like mice can help to experimentally untangle these interacting components and their underlying mechanisms. Mice have a rich communication system of vocal signals of different valence, which are processed through some of the same neural mechanisms as in human subjects. For example, male mice respond with greater approach behaviors to the playback of female squeaks when these are paired with the contextual odor of female urine, (indicating a reproductive opportunity) than the odor of cat fur (indicating a predatory threat; Grimsley et al., 2013). Neurons in the basolateral amygdala likewise show different patterns of responses to the same squeaks in the two different olfactory contexts (Grimsley et al., 2013). Affective brain regions in other animal models also show preferential responses to vocal signals than to neutral sounds or silence (Schubloom and Woolley, 2016; Salles et al., 2022). In mice, appetitive versus consummatory phases of opposite-sex interaction also show different types of vocal usage in conjunction with non-vocal context. The earlier stages of an opposite-sex interaction are characterized by a high production of non-harmonic USVs by males, and BBVs/squeaks by females that are paired with male-directed kicks, while later phases contain a higher proportion of harmonic USVs, and BBVs that are paired with male mounting behavior (Finton et al., 2017; Matsumoto and Okanoya, 2018). The split-cage assay allows us to address a situation in which rejective signals, BBVs, are presented within an opposite-sex context that engages high levels of male behavior and courtship.

The mouse model also allows investigation of the regulation of valenced vocal signals by serotonin (Wöhr et al., 2015). The serotonergic system is particularly interesting to explore in terms of its sensitivity to social cues, but also because it regulates multiple interacting sensory, affective, and cognitive neural systems, providing a possible mechanistic link among these (e.g., Hurley et al., 2004; Hamon and Blier, 2013; Seyedabadi et al., 2014; Jacob and Nienborg, 2018; Sizemore et al., 2020; Bishop et al., 2021; Steinbusch et al., 2021). The manipulation of serotonin can influence speech perception in older human subjects, improving the ability to identify a target from competing stimuli (Cruz et al., 2004; Gopal et al., 2005). Serotonin can also influence the perception of the sometimes extremely valenced auditory perception of tinnitus, although serotonergic manipulations may decrease or increase subjective tinnitus perception (Cruz et al., 2004; Oishi et al., 2010; Miller, 2016). A clue that serotonin could be involved in the interaction of hearing loss, aging, and social isolation is that this system shows plasticity in its infrastructure, from axon density through receptor expression, in response to both noise-induced hearing loss and social isolation (Papesh and Hurley, 2012; Smith et al., 2014; Keesom et al., 2018; Davis et al., 2021). Even more intriguingly, the plasticity associated with these factors is not limited to the auditory system. Early life social isolation is well-known for its effects on the anatomy and function of the serotonergic system in a wide range of brain regions (Lukkes, 2009). Even noise-induced hearing loss can result not only in decreased serotonergic density within the auditory system, but also further afield in regions including the hypothalamus, striatum, and frontal cortex (Li et al., 2019). These findings raise the possibility that serotonin, or other neuromodulatory systems, could be a mechanism involved in linking events like hearing loss or social isolation to later increased incidence of dementia and depression (Keesom and Hurley, 2020). Animal models, and behavioral assays assessing social responses to vocal signals, provide valuable tools to experimentally address these questions of the affective mechanisms of communication and communicative dysfunction.

## Data availability statement

The original contributions presented in the study are included in the article/Supplementary material, further inquiries can be directed to the corresponding author.

## Ethics statement

The animal study was approved by the Bloomington Institutional Animal Care and Use Committee. The study was conducted in accordance with the local legislation and institutional requirements.

## Author contributions

KH: Conceptualization, Data curation, Formal analysis, Investigation, Methodology, Writing—original draft, Writing—review and editing. LH: Conceptualization, Data curation, Formal analysis, Funding acquisition, Methodology, Project administration, Resources, Supervision, Writing—original draft, Writing—review and editing.

## Supplementary material

Supplementary Table 1 Correlations between male and female investigation times across time periods.
